# Ca^2+^ permeation and/or binding to Ca_V_1.1 fine-tunes skeletal muscle Ca^2+^ signaling to sustain muscle function

**DOI:** 10.1186/s13395-014-0027-1

**Published:** 2015-01-29

**Authors:** Chang Seok Lee, Adan Dagnino-Acosta, Viktor Yarotskyy, Amy Hanna, Alla Lyfenko, Mark Knoblauch, Dimitra K Georgiou, Ross A Poché, Michael W Swank, Cheng Long, Iskander I Ismailov, Johanna Lanner, Ted Tran, KeKe Dong, George G Rodney, Mary E Dickinson, Christine Beeton, Pumin Zhang, Robert T Dirksen, Susan L Hamilton

**Affiliations:** Department of Molecular Physiology and Biophysics, Baylor College of Medicine, One Baylor Plaza, Houston, TX 77030 USA; Department of Pharmacology and Physiology, University of Rochester Medical Center, 601 Elmwood Avenue, Rochester, NY 14642 USA

**Keywords:** Ca_V_1.1, CaM kinase II, Fatigue, Fiber type, Protein synthesis and Skeletal muscle

## Abstract

**Background:**

Ca^2+^ influx through Ca_V_1.1 is not required for skeletal muscle excitation-contraction coupling, but whether Ca^2+^ permeation through Ca_V_1.1 during sustained muscle activity plays a functional role in mammalian skeletal muscle has not been assessed.

**Methods:**

We generated a mouse with a Ca^2+^ binding and/or permeation defect in the voltage-dependent Ca^2+^ channel, Ca_V_1.1, and used Ca^2+^ imaging, western blotting, immunohistochemistry, proximity ligation assays, SUnSET analysis of protein synthesis, and Ca^2+^ imaging techniques to define pathways modulated by Ca^2+^ binding and/or permeation of Ca_V_1.1. We also assessed fiber type distributions, cross-sectional area, and force frequency and fatigue in isolated muscles.

**Results:**

Using mice with a pore mutation in Ca_V_1.1 required for Ca^2+^ binding and/or permeation (E1014K, EK), we demonstrate that Ca_V_1.1 opening is coupled to CaMKII activation and refilling of sarcoplasmic reticulum Ca^2+^ stores during sustained activity. Decreases in these Ca^2+^-dependent enzyme activities alter downstream signaling pathways (Ras/Erk/mTORC1) that lead to decreased muscle protein synthesis. The physiological consequences of the permeation and/or Ca^2+^ binding defect in Ca_V_1.1 are increased fatigue, decreased fiber size, and increased Type IIb fibers.

**Conclusions:**

While not essential for excitation-contraction coupling, Ca^2+^ binding and/or permeation via the Ca_V_1.1 pore plays an important modulatory role in muscle performance.

**Electronic supplementary material:**

The online version of this article (doi:10.1186/s13395-014-0027-1) contains supplementary material, which is available to authorized users.

## Background

Excitation-contraction coupling (ECC) in skeletal muscle involves a mechanical interaction between the L-type voltage-dependent Ca^2+^ channel (Ca_V_1.1) and the sarcoplasmic reticulum (SR) Ca^2+^ release channel (the ryanodine receptor, RyR1). Although Ca^2+^ entry through Ca_V_1.1 is not required for skeletal muscle ECC [1], Ca_V_1.1 opens after the initial voltage-gated release event to allow Ca^2+^ to both bind and permeate the channel pore [2]. Like other voltage-dependent Ca^2+^ channels, Ca_V_1.1 undergoes multiple conformational changes driven by membrane depolarization and/or Ca^2+^ movement (and/or binding) through the channel [3,4]. The opening of Ca_V_1.1 actually occurs after the opening of RyR1 [5], and Ca_V_1.1 has been suggested to facilitate RyR1 closing [6]. Although there is only limited understanding of the role of Ca^2+^ permeation via Ca_V_1.1, this influx has been suggested to regulate clustering of nicotinic acetylcholine receptors at the neuromuscular junction [7-9] and muscle plasticity [10].

Alterations in Ca^2+^ binding and permeation through Ca_V_1.1 contribute to the pathological development of several muscle diseases [11-14]. Mutations in both RyR1 and Ca_V_1.1 are associated with malignant hyperthermia (MH) in humans. The Ca_V_1.1 mutations either enhance Ca^2+^ influx, increase the Ca^2+^ sensitivity of RyR1, and/or destabilize a closed state of RyR1. At least one of these MH mutations (R174W) ablates the L-type current and increases the sensitivity of RyR1 to caffeine, but does not alter ECC, demonstrating that the disease mechanism involves a distinct role for Ca_V_1.1 [15]. Together, these findings suggest a more extensive role for Ca^2+^ permeation via Ca_V_1.1 in muscle function than what is presently understood.

In skeletal muscle, as in most cells, Ca^2+^ regulates multiple signaling pathways depending on the amplitude, frequency, duration, and location of the Ca^2+^ signal (reviewed by Tavi and Westerblad [16]). CaMKII is a major integrator of local Ca^2+^ signals that exhibits a privileged interaction with L-type channels (Ca_V_1.x) due to its ability to cluster nearby and, upon Ca^2+^ binding, to interact with Ca_V_1.x channels [17]. Ca_V_1.x channels prevail over other Ca^2+^ influx channels with respect to transducing excitation-transcription coupling (ETC) [18,19]. Hudmon *et al*. [17] demonstrated that CaMKII tethers to Ca_V_1.2 and phosphorylates the carboxy-terminal tail of the α_1_ subunit to increase Ca^2+^ influx. The interaction of CaMKII with Ca_V_1.2, in turn, facilitates CaMKII auto-phosphorylation.

To directly delineate the role of Ca^2+^ permeation through Ca_V_1.1 in skeletal muscle function, we created a mouse with a Ca^2+^ binding and permeation defect in Ca_V_1.1. We demonstrate that this Ca_V_1.1-mediated pathway utilizes the flexibility of Ca^2+^ signaling (location, frequency, duration, and amplitude) to regulate CaMKII and calcineurin activation, thereby enhancing SR Ca^2+^ store refilling and protein synthesis to modulate fatigue susceptibility, muscle size, and fiber type distribution.

## Methods

### Materials

N-benzyl-p-toluene sulfonamide (BTS) was purchased from Tocris Biosciences (Bristol/UK, England), latrunculin A from AdipoGen (San Diego/California, United States), and 4-chloro-m-cresol (4CmC) from Pfaltz & Bauer (West Chester/Pennsylvania, United States). The calcium dyes fura-2 AM and mag-fluo-4 AM were purchased from Invitrogen (Grand Island/New York, United States). Molecular weight marker for western blotting was purchased from GenDEPOT (Barker/TX, United States). Every other reagent including Insulin, KN-92, KN-93, and the myristoylated autocamtide 2-related inhibitory peptide (AIP) were purchased from Sigma-Aldrich (St. Louis, United States).

### Animals

In this study, male mice between six and 12 weeks old were used (The mice were generated by the Hamilton lab and backcrossed with the C57BL/6 J mice obtained from Jackson’s Laboratory (Bar Harbor/Maine, United States)), unless otherwise indicated. All mice were housed at room temperature with a 12:12 hour light-dark cycle and provided with food and water *ad libitum*. All procedures were approved by the Animal Care Committees at Baylor College of Medicine (Texas, United States) and the University of Rochester (New York, United States).

### Creation of E1014K mice

The EK mutation was engineered on a genomic fragment of about 500 bp containing the exon-encoding residue E1014. A tetracycline resistance gene cassette (tet, for bacteria selection) and a neomycin cassette (neo, for ES cell selection) were inserted into the middle of the engineered fragment, flanked by about 250 bp homologies to Ca_V_1.1. This selection marker-containing fragment was used to isolate a larger Ca_V_1.1 genomic clone from a mouse 129 phage library via homologous recombination in *Escherichia coli* [20]. Several clones were isolated and we chose one with an appropriate length of homologies on each side of the two cassettes for electroporation into AB2.2 ES from the 129SvEv cells. Recombinant ES clones were identified using southern blot analysis, and one of the clones was injected into blastocysts derived from C57BL/6 J mice to produce chimeras. The targeted allele was germ-line transmitted and the two selection cassettes were removed through crosses with Meox2-Cre mice [21].

To expedite the transfer of the E1014K mutation from the 129SvEv mouse sub-strain background to a congenic C57BL/6 J background, speed congenics were used, in addition to conventional backcrossing. We identified 92 microsatellites of maximal base-pair length disparity between the 129SvEv and C57BL/6 J strains. These microsatellites were chosen to representatively span the entire genome and show distinct electrophoretic separation. Primer pairs were selected to PCR amplify the chosen microsatellites to be resolved by electrophoresis in Spreadex EL300 Wide Mini Gels (Elchrom Scientific, Cham, Switzerland). Finally, we compared 129SvEv and C57BL/6 J DNA standards to DNA from our backcrossed mice, and selected mice with the most sequence homology to C57BL/6 J DNA to be used in the next backcross. This method both ensured congenicity between the wild-type (WT) and EK mutant mice and provided a means to more quickly begin experimentation.

### Isolation of flexor digitorum brevis muscle fibers

Skeletal muscle fibers were isolated from the flexor digitorum brevis (FDB) muscle obtained from WT and EK mice as described [22].

### Whole-cell patch clamp recordings of Ca_V_1.1 currents

The whole-cell patch clamp technique was used to assess Ca_V_1.1 currents (I_Ca_) in FDB fibers isolated from WT and EK mice. FDB fibers were bathed in an external recording solution containing (in mM): 157 TEA-methanesulfonate, 2 CaCl_2_, 10 HEPES, 0.5 anthracene-9-carboxylic acid (9-AC), and 0.1 BTS, at pH 7.4, adjusted with TEA-OH. The patch pipette internal solution contained (in mM): 140 Cs-methanesulfonate, 10 HEPES, 20 Na-EGTA, and 4 MgCl_2_, at pH 7.4, adjusted with CsOH. All reagents here were purchased from Sigma Aldrich (St. Louis/Missouri, United States). The patch pipette resistance when placed in the external solution was between 0.6 and 1.0 Mohm. Fibers were voltage-clamped at a holding potential of −80 mV. Series resistance was compensated up to 80%. Data were sampled every 120 μs and filtered using a low pass Bessel filter (Axon Instrument, Jakarta/Selatan, Indonesia) with a 2 kHz cut-off frequency. I_Ca_ was activated by 200 ms depolarizing pulses ranging from −40 mV to +60 mV in 10 mV increments delivered every 10 seconds. Ca_V_1.1 current-voltage relationships (I_Ca_-V) were obtained from peak currents measured during each depolarization normalized to cell capacitance and plotted against the corresponding test potential. I_Ca_-V data were then fitted by the following modified Boltzman equation:

1 $$ \left(\mathrm{V}\right) = {\mathrm{G}}_{\max }*\left({\mathrm{V}\hbox{-} \mathrm{V}}_{\mathrm{rev}}\right)/\left(1 + \exp \left[\left({\mathrm{V}}_{0.5}\hbox{--}\ \mathrm{V}\right)/{\mathrm{k}}_{\mathrm{g}}\right]\right) $$

where G_**max**_ is the maximal L-channel conductance, V is test potential, V_**rev**_ is the L-channel reversal potential, V_**0.5**_ is the potential for half-maximal activation of G_**max**_, and k_**g**_ is a slope factor.

Ca_V_1.1 currents were analyzed using Igor Pro 6 (Lake Oswego, Oregon, United States) and Clampfit 9 (Sunnyvale, California, United States) software.

### Measurements of electrically-evoked Ca^2+^ release in flexor digitorum brevis muscle fibers stimulated during a single twitch

Acutely isolated FDB fibers were loaded for 20 minutes at room temperature with 4 μM mag-fluo-4 AM in a Kreb’s Ringer solution containing (in mM): 146 NaCl, 5 KCl, 2 CaCl_2_, 1 MgCl_2_, and 10 HEPES, at pH 7.4. Fibers were then washed and incubated for 20 minutes in dye-free Ringer’s solution supplemented with 20 μM BTS, a skeletal muscle myosin inhibitor, to block contraction. Mag-fluo-4 AM-loaded FDB fibers were excited at 480 ± 15 nm and fluorescence emission detected at 535 ± 20 nm was collected at 10 kHz using a photomultiplier system. Electrical field stimulation (8 V, 1 ms, and 10 stimuli at 1 Hz) was elicited using a glass electrode placed adjacent to the cell of interest. Peak changes in mag-fluo-4 fluorescence for all 10 stimuli were measured as (F_max_-F_0_)/F_0_ and then averaged to generate a single peak value for each fiber. The rate of mag-fluo-4 fluorescence decay for the second, third, and fourth twitches for each fiber was fitted to a first order exponential function and the resulting amplitude and tau values were averaged.

### Mn^2+^ quench measurements

Mn^2+^ quench of fura-2 emission was measured in myotubes loaded with 5 μM fura-2 AM for 1 h at 37°C in Kreb’s Ringer solution. Briefly, prior to Mn^2+^ quench measurements, myotubes (primary cultured cells from muscle of mice) were treated with 100 μM ryanodine to block RyR1-mediated Ca^2+^ release during subsequent KCl application. Fura-2-loaded myotubes were excited at the experimentally determined fura-2 isosbestic point (362 nm) and emission monitored at 510 nm during perfusion of 50 mM KCl in the presence of 0.5 mM Mn^2+^. Maximum rates of fura-2 quench during KCl application were determined and evaluated for statistical significance.

### Calcium imaging in confocal line scan mode in flexor digitorum brevis muscle fibers stimulated with a single 50 Hz train

To monitor Ca^2+^ release during electrical stimulation, FDBs fiber were loaded with 5 μM of mag-fluo-4 AM for 30 minutes at room temperature in the presence of 20 μM of BTS. Loaded FDBs were placed on the stage of a confocal microscope with an adapted perfusion system (tyrode with 20 μM BTS at 0.5 mL/min) and imaged in line scan mode using the 20x objective (EC Plan-Neofluar) mounted in the confocal microscope (Zeiss LSM 510 meta, California, United States), one line was acquired every 1.15 milliseconds (3.66 μsec/pixel time) using the 488 nm excitation laser and the LP 505 emission filter (Zeiss, California, United States). FDBs were stimulated with 50 square electrical pulses (200 μsec duration) at 50 Hz and the produced florescence transients was normalized (F/F_0_) and plotted.

### Measurement of Ca^2+^ transients during repetitive stimulation with 100 Hz trains

Isolated FDB fibers were loaded with 5 μM of mag-fluo-4-AM for 30 minutes at room temperature, followed by washout with fresh DMEM (Life technologies, NY, United States). Electrical stimulation was performed using two platinum wires placed at each side of the fiber oriented longitudinally and fatigue was induced with uninterrupted application of electrical trains (100 Hz, 250 ms, every 1.5 seconds; 0.17 duty cycle) for 300 seconds. For evaluation of RyR1-releasable SR Ca^2+^ store content, 1 mM of 4CmC was perfused at 3.25 ml/min, applied after 60 trains of electrical stimulation. Mag-fluo-4 fluorescence was collected at 20 Hz. Data were collected and analyzed using Metafluor version 6.2 software (Molecular Devices, California, United States).

### Western blotting

Muscles were homogenized and lysed in ice-cold RIPA buffer consisting of (mM): 25 Tris pH 7.6, 150 NaCl, 1 Na_3_VO_4_, 10 NaPyroPO_4_, 10 β-glycerophosphate, 10 NaF, PMSF, protease inhibitor cocktail (Santa Cruz), 1% NP40, 1% sodium deoxycholate, and 0.1% SDS (Every reagents came from Sigma Aldrich, St. Louis, United States). Equal amounts of total protein from whole muscle lysates were resolved by electrophoresis, transferred to PVDF (Millipore, Billercia, United States) membrane and western blot analyses were performed using antibodies shown in Additional file 1: Table S1. LI-COR IRDye™ infrared dyes were used as secondary antibodies and immunoreactive bands were visualized using the Odyssey Infrared Imaging System (LI-COR) (LI-COR Inc, Lincoln, United States). To allow the use of data from multiple western blots, the fluorescent band intensity of each band within a single western was first normalized to GAPDH (Glyceraldehyde 3-phosphate dehydrogenase) as a loading control and then calculated as %WT average from that specific western blot. Data were then pooled to give %WT ± SEM.

### Co-localization studies and single fiber immunocytochemistry

Single FDB fibers plated on glass slides were fixed with 2% paraformaldehyde in 0.1 M phosphate buffer (PB) (21.6 mM Na_2_HPO_4_ and 81.4 mM NaH_2_PO_4_, pH7.2) for one hour at room temperature, washed twice with phosphate buffered saline (PBS) (3.8 mM NaH_2_PO_4_, 16.2 mM Na_2_HPO_4_, 150 mM NaCl, pH 7.4), permeabilized, and blocked in PBS containing 0.5% Triton X-100 and 5% BSA overnight at 4°C (All reagents come from Sigma Aldrich as described in Materials). Primary antibodies diluted in PBST (PBS containing 0.5% TX-100) were added to slides and incubated overnight at 4°C. After washing twice with PBS, Alexa-fluor conjugated antibodies were added. Fibers were washed three times with PBS for 10 minutes each and mounted in Fluoromount-G (SouthernBiotech, Birmingham, United States). Fibers were imaged using a Zeiss LSM 510 META confocal microscope with a 100x/1.30NA oil lens, HeNe 543 nm laser, and Argon 458,477,488,514 laser (Zeiss, California, United States).

### Proximity ligation assay

We used proximity ligation assays (PLAs) to identify proteins that are within 40 Å of each other. PLA was performed on single FDB fibers plated on glass disks. Fibers were kept at 37°C in a 95% O_2_-5% CO_2_ incubator in DMEM solution supplemented with 10% FBS. Fibers were then fixed with 2% paraformaldehyde in 0.1 M PB and incubated with the primary antibodies. The PLA was performed with the Duolink kit (Olink Biosciences, Uppsala, Sweden) according to the instruction of the user manual using an anti-goat MINUS PLA and anti-rabbit PLUS PLA probes and the orange detection reagent (Cy3) (Olink Biosciences, Uppsala, Sweden). Fibers were imaged with confocal microscopy (Zeiss LSM 510 META, with a 100x/1.30NA oil lens and HeNe 543 nm laser). For analysis, Z stacks were projected and saved as a single image. The positive spots were counted with Image J (8-bit images filtered with a Gaussian Blur filter (Rasband, W.S Image J, U.S. National Institutes of Health, Bethesda, Maryland, United States), σ = 1, and same threshold per set adjusted at 15-30). The number of spots counted was normalized to the area of the fiber estimated from the width and length of the fiber in the image. For each set of experiments, the average counts of WT fibers (control) were set to 100% and the number of spots in each experimental condition was calculated as percentage change.

### Insulin treatment

Eight-week-old WT and EK mice were fasted for 12 hours and then given an intraperitoneal injection of insulin (1 U/kg) diluted in saline. Control mice were injected with saline. After 7.5 minutes mice were sacrificed and muscles (soleus and EDL (Extensor Digitorum Longus)) were isolated, frozen in liquid nitrogen, and stored at −80°C until use. Muscle levels of pAkt1/2 and pGSK3β in the presence and absence of insulin were measured as described in Butler *et al*. [23].

### Detection of puromycin-labelled proteins

For measurement of protein synthesis we used an *in vivo* SUnSET technique [24,25]. Briefly, mice (13 weeks of age) were food deprived for eight hours. Propofol (18 μl/g) (Abbott Laboratories, North Chicago, United States) was administered via an intraperitoneal injection 15 minutes before the puromycin injection. The mice were then given an intraperitoneal injection of puromycin (0.04 μmol/g BW) and sacrificed 35 minutes later. At 10 minutes before sacrificing, insulin or saline (control) was administered via intraperitoneal injection. Muscles were isolated, homogenized, and prepared for western blotting with anti-puromycin antibody. For normalization to total protein, the same western blots were stained with Swift Membrane Stain™ kit (G-Biosciences, St. Louis, United States).

### Ras activity

Ras activity was measured using a Ras activity assay kit (Cytoskeleton, Denver, United States). Briefly, muscle was lysed in buffer and protein concentration was measured. Raf-Ras binding domain (RBD) beads (50 μl) were added to the muscle lysates (total 500 μl of 2 mg/ml lysates) and the mixture was incubated at 4°C on a rotator for one hour. After incubation, Raf-RBD beads were pelleted by centrifugation at 5,000 × g at 4°C for one minute and washed with wash buffer. The bound active Ras was eluted in the two × sample buffer by boiling for three minutes. Eluted protein was run on 12% gel, transferred to PVDF membrane, and immunoblotted with Ras-specific antibody. The westerns were normalized to the amount of GAPDH in the muscle lysates (60 μg).

### Sarco/endoplasmic reticulum Ca^2+^-ATPase activity

Sarco/endoplasmic reticulum Ca^2+^-ATPase (SERCA) activity in tissue homogenates was performed as described [26,27].

### Electrical stimulation of isolated muscle for signaling changes

To assess stimulation-induced changes in signaling pathways we used the method of Sakamoto *et al*. [28]. Intact soleus and EDL muscles were removed and suspended between a force transducer and stationary anchor within a test chamber filled with warmed (35°C) Kreb’s Ringer solution (KRS) oxygenated with a 95/5% mixture of O_2_/CO_2_, as above. After a 30 minute resting equilibration period, muscles to be stimulated underwent a fatigue protocol (100 Hz, 200 ms train duration, one second intervals) for five minutes per muscle. At the completion of the stimulation protocol, rested and stimulated muscles were immediately frozen in liquid N_2_ and stored at −80°C. For muscles treated with KN-93, the drug was added to the chamber at the start of the 30 minutes equilibration period, at a final concentration of 5 μM.

### Muscle force frequency and fatigue

Intact soleus and EDL muscles were removed and immediately immersed in incubation medium comprised of KRS (oxygenated with a 95/5% mixture of O_2_/CO_2_. Muscles were tied with sutures at the musculotendinous junction and suspended between a force transducer and stationary anchor within a test chamber filled with warmed (35°C), oxygenated incubation medium. After a 20 minutes rest to allow mounted muscles to equilibrate, muscle optimal length (*l*_*o*_) was determined via single-twitch force generation measurements. Next, force frequency measurements were obtained at *l*_*o*_ using frequencies from 15 to 300 Hz at 200 ms/train followed by a fatigue protocol performed over five minutes per muscle. The specific fatigue protocol for each muscle used was for the soleus: 15 Hz, 200 ms duration, one second intervals) and for EDL: 60 Hz, 200 ms duration, one second intervals. Muscle stimulation occurred within the test chamber using platinum electrodes attached to a Grass S48 stimulator and recorded within Chart5 (version 5.2) software (eDAQ Inc, Colorado Springs, United States).

### Fibertyping with cryosections and immunostaining

Skeletal muscles (soleus and EDL) were dissected, embedded in OCT compound (Tissue-Tek, Torrance, United States), and frozen in 2-methylbutane (Sigma Aldrich, St. Louis, United States) precooled in liquid nitrogen. The frozen muscles were sectioned with 10-μm thickness using a SHANDON cryostat microtome (Thermo Electron Corporation, Madison, United States). Immunofluorescent staining was performed using specific antibodies against myosin heavy chain I (MHCI), IIa (MHCIIa), and IIb (MHCIIb) (DSHB, Iowa City, USA). Briefly, sections were rehydrated with PBS for 10 minutes, followed by incubation at 4°C overnight with MHCI (BA-F8, IgG_2b_), MHCIIa (SC-71, IgG_1_), and/or MHCIIb (BF-F3, IgG_M_) antibodies diluted 1:50 in PBS. After washing with PBS, muscle sections were incubated at room temperature for 90 minutes with isotype-specific AlexaFluor-594-conjugated goat anti-mouse IgG_2b_, AlexaFluor-488-conjugated goat anti-mouse IgG_1_, and AlexaFluor-594-conjugated goat anti-mouse IgG_M_ secondary antibodies diluted 1:200. After three consecutive washes with PBS, muscle slides were mounted with VECTASHIELD mounting media (Vector Laboratories, Burlingame, United States). Images were taken under a fluorescence microscope (Olympus America, Center Valley, United States). The relative numbers of the different fiber types were quantified and normalized by the total number of muscle fibers per field.

### Fiber cross-sectional area

For cross-sectional area (CSA) calculations, 10-μm-thick frozen sections were obtained from the mid-belly area of the soleus and EDL muscles. Sections were immunostained for fiber type as described in the previous section, imaged at 10x magnification through an Olympus DP70 camera (Olympus America, Center Valley, Pennsylvania, United States), and saved in .tif format. Saved images were then imported into Photoshop CSE version 10.0 (Adobe Systems, San Jose, California, United States) for analysis. First, measurement scale was established by tracing a within-image scale bar (μm). Next, myofiber CSA was measured by tracing the external border of individual myofibers using the Magnetic Lasso tool. Myofibers exhibiting evidence of tears or processing artifacts were excluded from the analysis. Recorded measurements were then exported into Excel for analysis, with resulting CSA values reported in μm^2^.

### Statistical analyses

We performed a statistical analyses of two groups using the Student’s *t*-test. *P* <0.05 was considered to be statistically significant. *P <0.05, **P <0.01, and ***P <0.001 were used to indicate statistical significance.

## Results

### Creation of EK mice

To explore the role of Ca^2+^ influx via Ca_V_1.1, we created mice with a knock-in mutation (E1014K or EK) in the pore region of Ca_V_1.1. The mutation of a glutamate to lysine residue in the repeat III pore region of Ca_V_1.x abolishes both Ca^2+^ binding to this site [29] and divalent permeation through the channel without altering ECC [2]. The targeting construct and data confirming the mutation are shown in Additional file 2: Figure S1. Speed congenics were used to obtain mice on a clean C57BL/6 J background. The mice were homozygous viable with no immediately obvious changes in phenotype (see below).

### Effects of the EK mutation on myofibrillar Ca^2+^ handling

**Figure 1:**
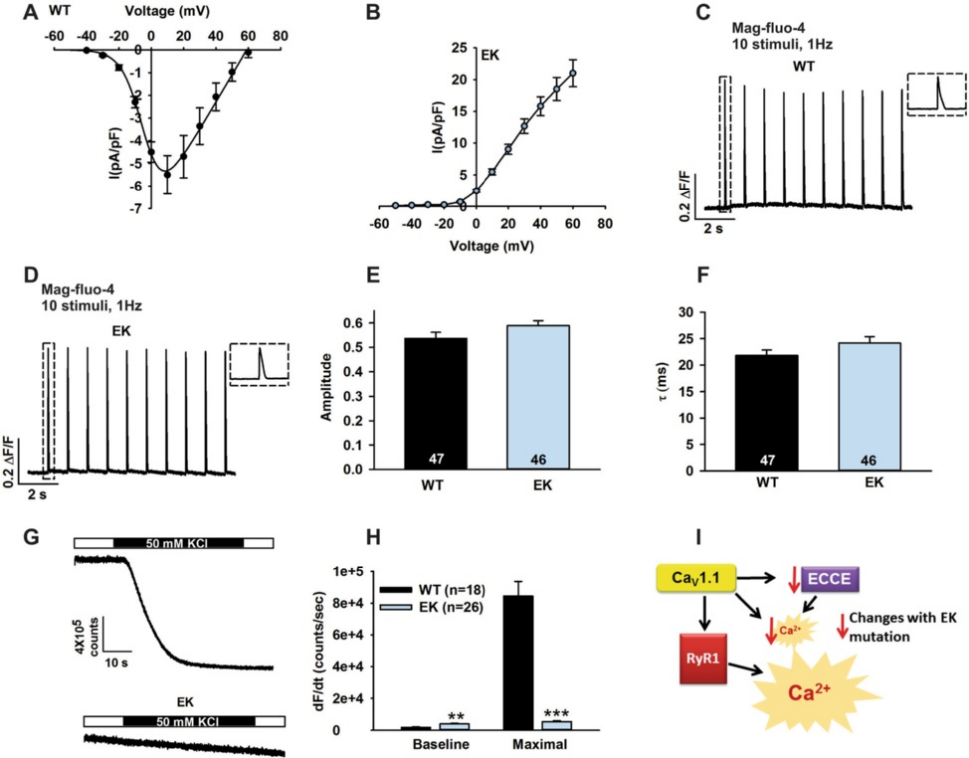
**Effects of the EK mutation on Ca**_**V**_ **1.1 currents, ECC, and ECCE.** Voltage dependence of peak Ca_V_1.1 current density for FDB fibers from **(A)** WT mice (n = 5) and **(B)** EK mice (n = 9). **(C and D)** Representative traces of electrically-evoked mag-fluo-4 transients in FDB fibers obtained from **(C)** WT and **(D)** EK mice. Insets: The first transient for each condition on an expanded time scale in FDB fibers obtained from WT and EK mice. **(E)** Average amplitude and **(F)** decay constant of the recovery phase in FDB fibers from WT and EK mice. **(G)** Representative Mn^2+^ quench of fura-2 fluorescence in myotubes from WT and EK mice. **(H)** Average rate of Mn^2+^ quench in myotubes from WT and EK mice. **(I)** Scheme of ECC changes altered in EK muscle. Data are shown as mean ± SEM. **P <0.01 and ***P <0.001.

To confirm that the EK mutation abolishes Ca^2+^ influx through Ca_V_1.1, we compared whole-cell Ca^2+^ currents (Additional file 3: Figure S2A and B) and average Ca_V_1.1 current-voltage relationships (Figure 1A and B) in single FDB fibers from EK and WT mice. The EK mutation abolished inward L-type Ca^2+^ currents, but permitted outward monovalent Cs^+^ currents. The permeation of EK channels to monovalent cations (for example, Cs^+^ and Na^+^; [30]) is unlikely to alter resting membrane because the EK monovalent cation conductance is only activated at depolarized potentials. Also, since the activation kinetics of EK channels is slow [2,30] relative to the duration of the skeletal muscle action potential, Na^+^ flux through EK channels should be minimal, and thus unlikely to alter action potential properties or intracellular Na^+^ levels (approximately 10 mM). The magnitude and decay kinetics of voltage-gated Ca^2+^ transients (assessed using a low affinity Ca^2+^ indicator, mag-fluo-4 AM) elicited by a low frequency train of electrical stimulation (1 Hz) were not significantly different between FDB fibers from WT and EK mice (Figure 1C-F), demonstrating that ECC was not altered by the EK mutation.

We used a well-established Mn^2+^ quench of fura-2 fluorescence assay to determine the effect of the EK mutation on excitation-coupled Ca^2+^ entry (ECCE) [31]. In WT myotubes, membrane depolarization induced by the addition of 50 mM KCl opens Ca_V_1.1 channels, providing a pathway for Mn^2+^ entry to quench fura-2 fluorescence (Figure 1G, upper trace), with the maximum slope of KCl-induced Mn^2+^ quench directly reflecting Mn^2+^ entry through Ca_V_1.1 channels (Figure 1H). Since the EK mutation in the Ca_V_1.1 pore abolishes divalent ion permeation through Ca_V_1.1 channels, KCl-induced Mn^2+^ quench (ECCE) is absent in myotubes from EK mice (Figure 1G, lower trace), consistent with ECCE reflecting Ca^2+^ entry via Ca_V_1.1, as suggested by Bannister *et al*. [32]. The EK fibers also showed a small but significant increase in baseline Ca^2+^ influx (Figure 1H).

**Figure 2:**
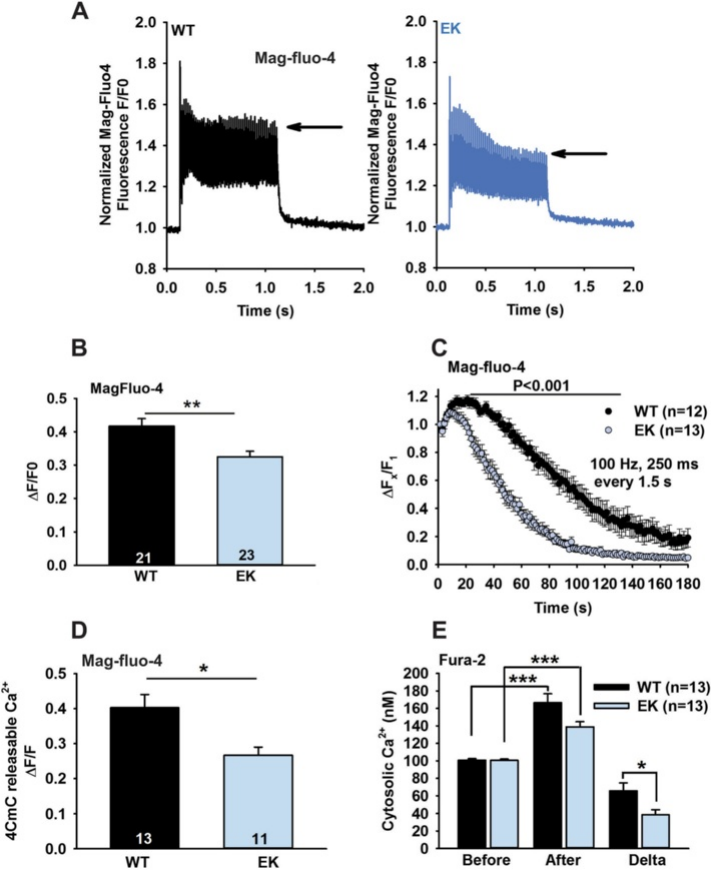
**Effects of repetitive stimulation. (A)** Representative traces for mag-fluo-4 fluorescence in WT and EK FDB fibers subjected to a single 50 Hz train of stimulation for one second and the response was acquired in the line scan mode of a confocal microscope. **(B)** Tetanic calcium response averaged from first to 50th peak (50 pulses for one second) and the calculated averaged response was plotted. **(C)** Effects of fatiguing stimulation (100 Hz) on the amplitude of the Ca^2+^ transients in WT and EK fibers measured with mag-fluo-4. **(D)** Average amplitude of the 4CmC releasable Ca^2+^ stores after repetitive stimulation (100 Hz) measured with mag-fluo-4. **(E)** Cytosolic Ca^2+^ concentrations measured with Fura-2 before and after electrical stimulation using the same stimulation protocol as in **(C)**. Values represent the average values over one second at 30 seconds after stimulation. Data are shown as mean ± SEM. *P <0.05, **P <0.01, and ***P <0.001.

While the data in Figure 1 indicate that ECC is not altered in EK fibers, we found significant differences in the amplitude of the Ca^2+^ transients during repetitive stimulation. During a single train of high frequency stimulation, the amplitude of the Ca^2+^ transients was lower in EK compared to WT fibers (Figure 2A and B). The EK mutation also increased the rate of decline of the amplitude of the Ca^2+^ transient during repetitive trains of high frequency stimulation (Figure 2C). After these repetitive trains of stimulation, readily releasable SR Ca^2+^ stores assessed with a maximal concentration (1 mM) of 4-chloro-m-cresol (4CmC) were significantly lower in EK compared to WT fibers (Figure 2D), suggesting greater Ca^2+^ store depletion in the EK fibers following repetitive stimulation. However, readily releasable SR Ca^2+^ stores were not different prior to electrical stimulation (data not shown).

We used a higher affinity Ca^2+^ indicator, fura-2 AM, to measure changes in cytosolic Ca^2+^ levels. Resting cytosolic Ca^2+^ levels did not differ between EK and WT fibers. However, following stimulation (same stimulation protocol as Figure 2C), cytosolic Ca^2+^ levels were higher in both EK and WT fibers (Figure 2E), but magnitude of the stimulation-induced increase in cytosolic Ca^2+^ was less in EK fibers. These findings suggest that Ca^2+^ permeation through Ca_V_1.1 facilitates refilling of SR Ca^2+^ stores either directly or indirectly. The decreased Ca^2+^ transient amplitude during repetitive stimulation in EK fibers could be due to an decrease in Ca^2+^ influx via either store-operated or ECCE [31].

**Figure 3:**
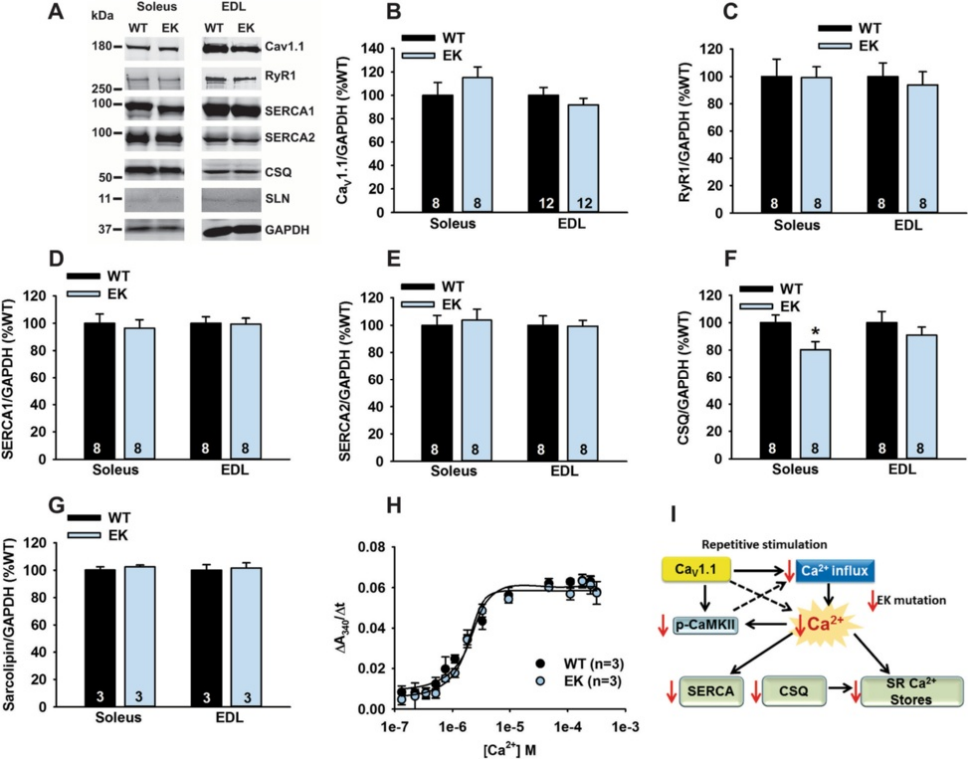
**Ca** ^**2+**^ **handling proteins. (A)** Representative western blot images of Ca_V_1.1, RyR1, SERCA1, SERCA2, calsequestrin (1 and 2), and sarcolipin. To allow the use of data in multiple western blots each band within a single western blot was normalized to GAPDH for that sample as a loading control and then normalized to the average WT values for that particular gel to give %WT. **(B)** Analysis of muscle levels of Ca_V_1.1 normalized to GAPDH. **(C)** Analysis of muscle levels of RyR1 normalized to GAPDH. **(D)** Analysis of muscle levels of SERCA1 normalized to GAPDH. **(E)** Analysis of muscle levels of SERCA2 normalized to GAPDH. **(F)** Analysis of muscle levels of CSQ normalized to GAPDH. **(G)** Analysis of sarcolipin normalized to GAPDH. **(H)** SERCA activity as a function of Ca^2+^ concentration. **(I)** Scheme of changes in Ca^2+^ handling proteins. Values are shown as mean ± SEM. *P <0.05.

Alterations in Ca^2+^ handling in EK muscle could also arise from changes in Ca^2+^ handling proteins. However, we detected no differences in the expression levels of Ca_V_1.1, RyR1, SERCA1, or SERCA2 (Figure 3A-E). We did, however, detect a decrease in calsequestrin (CSQ, antibody detects both CSQ1 and 2) in the soleus (Figure 3F). Sarcolipin levels were unchanged (Figure 3G). SERCA activity in muscle homogenates at a fixed Ca^2+^ concentration was not different between EK and WT muscle (Figure 3H). However, differences in cytoplasmic Ca^2+^ levels are likely to impact SERCA activity in intact fibers during repetitive stimulation.

### Pleiotropic effects of the EK mutation on Ca^2+^-sensitive pathways

**Figure 4:**
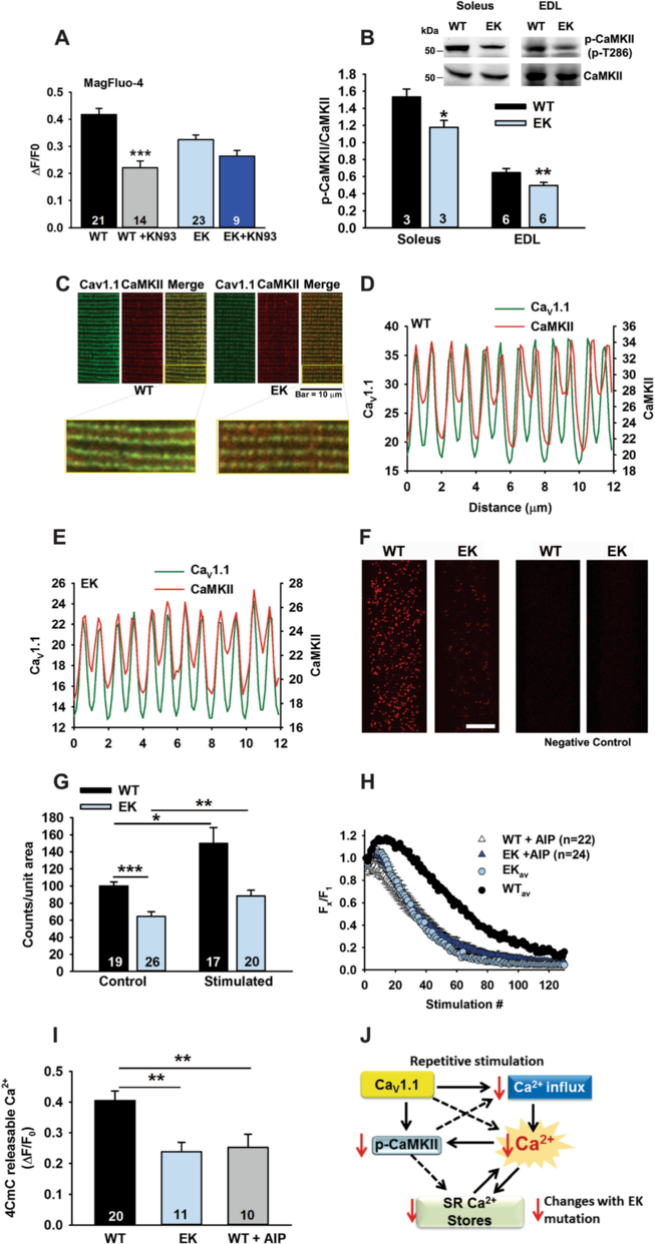
**Activity-dependent CaMKII translocation and activation. (A)** KN-93 decreases the height of the Ca^2+^ transient during repetitive stimulation. Tetanic calcium response averaged from first to 50th peak (50 pulses for one second) and the calculated averaged response was plotted as in Figure 2B. **(B)** Ratio of p-CaMKII to CaMKII in muscles of EK and WT mice (%WT). Inset: Representative western blot of p-CaMKII and CaMKII. **(C)** Representative immunocytochemistry images showing co-localization of CaMKII and Ca_V_1.1 in single WT and EK FDB fibers. **(D and E)** Representative line profiles of immunofluorescence for CaMKII and Ca_V_1.1 in **(D)** WT and **(E)** EK FDB fibers. **(F)** Representative images for the PLA assay confirming a close association of CaMKII with Ca_V_1.1. Scale bar = 20 μm. For negative control (right), normal rabbit IgG was used instead of CaMKII antibody. **(G)** Analysis of average spot density in the proximity ligation assay in fiber resting and electrically stimulated. Spots are analyzed in fibers from three mice of each genotype. **(H)** Effect of AIP on the amplitude of the Ca^2+^ transient with repetitive stimulation. **(I)** 4CmC-induced Ca^2+^ release post stimulation in the presence and absence of AIP. **(J)** Changes associated with the EK mutation in Ca_V_1.1. Values are shown as mean ± SEM. *P <0.05, **P < 0.01, and ***P < 0.001.

The decreased amplitude of the Ca^2+^ transient during repetitive stimulation in EK fibers is likely to impact multiple muscle Ca^2+^ signaling pathways. As shown in Figure 4A, KN-93 decreases the amplitude of the Ca^2+^ transient in WT but not EK fibers, suggesting a role for CaMKII. To evaluate the effects of the EK mutation on CaMKII, we first assessed the ratio of pT286-CaMKII to total CaMKII using western blotting. Auto-phosphorylation of CaMKII at T286 is associated with its constitutive activation [33]. We found that the ratio of p-CaMKII to CaMKII was decreased in the soleus and EDL of EK mice (Figure 4B). Since CaMKII is a known integrator of Ca^2+^ signals and has an interaction with L-type channels in other tissues [17], we used immunocytochemistry and proximity ligation assays (PLA) to determine if CaMKII was located close to Ca_V_1.1 in skeletal muscle FDB fibers. We found that a significant amount of CaMKII co-localizes with Ca_V_1.1 in both WT and EK fibers by immunocytochemistry (Figure 4C-E) and by PLA (Figure 4F and G). As assessed in the PLA assay, the interaction of Ca_V_1.1 and CaMKII was decreased in the EK compared to WT fibers under both resting and electrically stimulated conditions (Figure 4G). Thus, CaMKII is in close proximity to Ca_V_1.1 and this interaction is decreased by the permeation defect in Ca_V_1.1.

We next assessed the effects of inhibition of CaMKII in WT fibers on refilling of SR Ca^2+^ stores during repetitive stimulation. We used a specific CaMKII inhibitory peptide, AIP (myristoylated CaMKII auto-inhibitory peptide) [34], and found that CaMKII inhibition increased the rate of decline in the amplitude of the Ca^2+^ transients (Figure 4H,) and reduced post-stimulation 4CmC releasable Ca^2+^ stores (Figure 4I) in FDB fibers from WT mice. KN-93 (but not the inactive K-92 analog) also reduced the amplitude of the Ca^2+^ transients in WT fibers but had no effect in EK fibers (Additional file 3: Figure S2C and D and Figure 4H). These data suggest that SR Ca^2+^ store refilling during repetitive stimulation in WT fibers is enhanced by Ca_V_1.1-mediated activation of CaMKII, but this pathway is prevented by the EK mutation in Ca_V_1.1 (Figure 4J).

**Figure 5:**
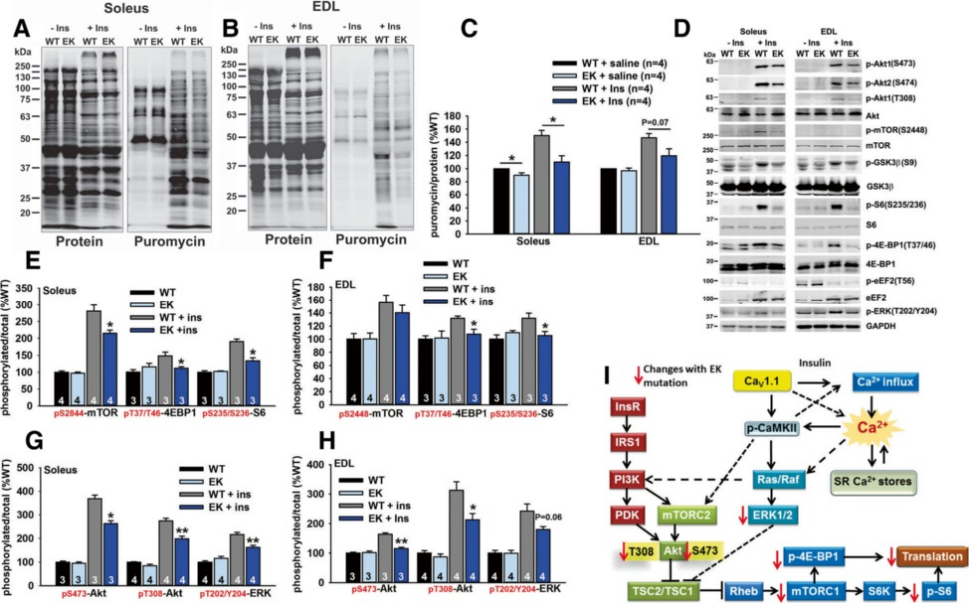
**Effect of EK mutation on growth signaling pathways. (A)** Representative anti-puromycin western blot for soleus muscle homogenates from EK and WT mice treated with insulin 25 minutes after puromycin. Also shown is a protein stain as a loading control. **(B)** Representative anti-puromycin western blot for EDL muscle homogenates from EK and WT mice treated with insulin 25 minutes after puromycin. Also shown is a protein stain as a loading control. **(C)** Analysis of anti-puromycin/protein in the soleus and EDL of saline and insulin-treated EK and WT mice. **(D)** Western blot for protein involved in protein synthesis as %WT average for each western blot using homogenates from the soleus and EDL muscles from mice treated with saline or insulin. **(E and F)** Analysis of indicated phospho protein to dephosphorylated proteins (plotted as %WT average) in soleus and EDL. **(G and H)** Analysis of indicated phospho protein to dephosphorylated protein (plotted as %WT average) in soleus and EDL. **(I)** Pathways altered by the EK mutation alter protein synthesis. Values are shown as mean ± SEM. *P <0.05 and **P <0.01.

Ca^2+^ is required for mTORC1 activation of p70 ribosomal S6 kinase 1 (S6K1) [35-39]. To determine if the absence of Ca^2+^ permeation through Ca_V_1.1 altered protein synthesis, we used the SUnSET technique [25] to assess protein synthesis in EK and WT muscle. Although we detected a small decrease in protein synthesis in the absence of insulin in the soleus, protein synthesis was significantly decreased in both the soleus and EDL muscle of EK mice treated with insulin compared to insulin-treated WT mice (Figure 5A-C). To further analyze protein synthesis pathways downregulated by the absence of Ca^2+^ permeation through Ca_V_1.1, we examined the phosphorylation status of several key regulators of protein synthesis. We found that pS2448 mTOR, p-T37/T46-4EBP1/4EBP1, and p-S235/S236-S6/S6 were reduced in the soleus and EDL of insulin-treated EK mice compared to insulin-treated WT mice (Figure 5D-F). To identify upstream events that regulate protein synthesis, we examined the levels of p-S473-Akt/Akt (phosphorylated by mTORC2) [40], pT308-Akt/Akt (phosphorylated by PDK1), and pT202/Y204-ERK1/2 and found that all of these phosphorylation events were decreased in both the soleus and EDL of EK mice treated with insulin compared to the corresponding muscle of insulin-treated WT mice (Figure 5D, G, and H). Hence the muscle response to insulin is blunted in the EK mice due to decreased mTORC2 and PDK1 phosphorylation of Akt and decreased ERK1/2 activity (Figure 5I). There are multiple Ca^2+^ and/or CaMKII sensitive step(s) upstream of mTORC2, PDK1, and ERK signaling, especially in the steps that lead to Ras activation.

**Figure 6:**
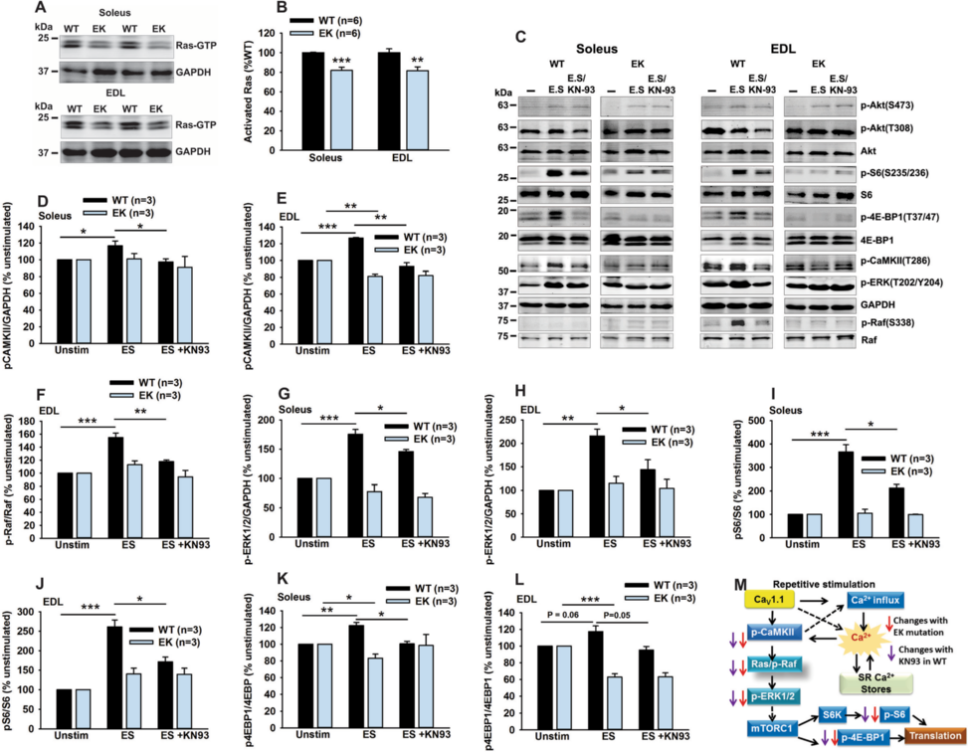
**Ca**_**V**_ **1.1/CaMKII modulates Ras/Raf/ERK1/2 signaling to increase mTORC1 signaling in response to electrical stimulation. (A)** Representative western blot for activated Ras (Ras-GTP) in Raf-RBD pull-down from WT and EK muscle. **(B)** Analysis of activated Ras (Ras-GTP) in Raf-RBD pull-down in muscle from WT and EK mice. **(C)** Electrical stimulation-induced changes in mTORC1 signaling. Representative western blots of isolated solei and EDL muscles subjected to a five-minute electrical stimulation (as described in Methods) in the presence and absence of KN-93 (5 μM). For all analyses the values for the western blots of individual bands from WT and EK muscle are each normalized to the values obtained in the absence of electrical stimulation. For statistical analyses the WT and EK are each compared to their unstimulated controls. **(D)** Analysis of pCaMKII/GAPDH in the soleus. **(E)** Analysis of pCaMKII/GAPDH in the EDL. **(F)** Analysis of p-Raf (a CaMKII target) in the EDL. **(G)** Analysis of pERK1/2/GAPDH in the soleus. **(H)** Analysis of pERK1/2/GAPDH in the EDL. **(I)** Analysis of pS6/S6 in the soleus. **(J)** Analysis of pS6/S6 in the EDL. **(K)** Analysis of p4EBP1/4EBP1 in the soleus. **(L)** Analysis of p4EBP1/4EBP1 in the EDL. **(M)** Summary of changes in signaling pathways. Values are shown as mean ± SEM. *P <0.05, **P <0.01, and ***P <0.001.

The data with and without insulin suggest that the effects of the EK mutation on insulin-stimulated protein synthesis involve both Akt phosphorylation (PDK1 and mTORC2) and ERK signaling. To assess Ras activation in EK and WT muscle, we pulled down activated Ras (GTP bound) with Raf-RBD-beads from soleus and EDL muscle and western blotted with RAS specific antibody to evaluate the amount of Ras that pulled down. As shown in Figure 6A and B, less GTP-bound Ras was pulled down in EK compared to WT muscle. To further assess the role of Ca^2+^ permeation and binding to Ca_V_1.1 and activation of CaMKII in the regulation of these pathways, we isolated soleus and EDL muscle from WT and EK mice, electrically stimulated in the presence and absence of KN-93 (an inhibitor of CaMKII) and again analyzed the pathways described in Figure 5. Electrical stimulation increased p-CaMKII/GAPDH, p-Raf/Raf (a CaMKII target), p-ERK1/2/GAPDH, p-S6/S6, and p-4EBP1/4EBP1 (Figure 6C-L), and these increases were less in the presence of the CaMKII inhibitor, KN-93. With the exception of p-Raf/Raf, all of these changes were detected in both soleus and EDL. Phosphorylated Raf was detected only at very low levels in the soleus. In contrast to the findings in WT muscle, no increases in p-CaMKII/GAPDH, p-Raf/Raf (a CaMKII target [41]), p-ERK1/2/GAPDH, p-S6/S6, and p-4EBP1/4EBP1 were detected in the EK muscle subjected to electrical stimulation.

We also examined the phosphorylation of Akt at S473 and T308 and found that phosphorylation of Akt at these sites were not significantly increased by electrical stimulation (Additional file 3: Figure S2E-H), suggesting that the signaling in response to repetitive stimulation is primarily through Ras/Raf activation of ERK1/2. Given these findings, we propose that Ca^2+^ permeation though or binding to Ca_V_1.1 activates CaMKII which, in turn, phosphorylates Raf to activate Ras/Raf and ERK1/2. ERK1/2 then activates mTORC1 (Figure 6M). This stimulation-dependent signaling pathway is strongly blunted in EK muscle.

Together, our data provide compelling evidence that the EK mutation in Ca_V_1.1 causes significant disruption to CaMKII and Ca^2+^-sensitive pathways in skeletal muscle, resulting in decreased SR store refilling and a decline in protein synthesis.

### Effects of EK mutation on muscle function

**Figure 7:**
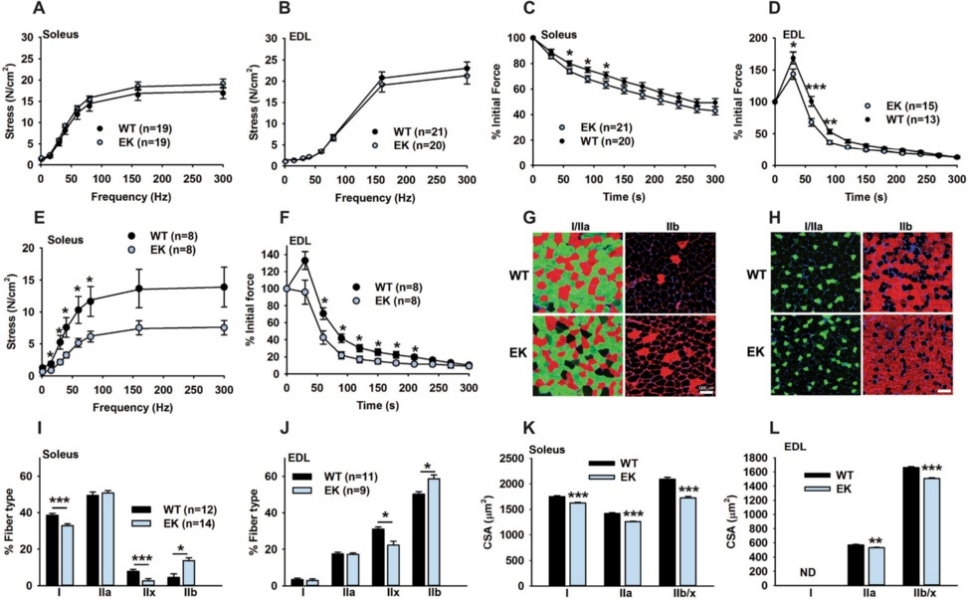
**Muscle function. (A)** Force frequency relationship for the soleus of eight-week-old mice. **(B)** Force- frequency relationship for the EDL of eight-week-old mice. **(C)** Fatigue plotted as % initial force in the soleus of eight-week-old mice. **(D)** Fatigue plotted as % initial force in the EDL of eight-week-old mice. **(E)** Force frequency relationship for the soleus of mice over nine months old **(F)** Fatigue plotted as % initial force in the EDL of mice over nine months old. **(G** and **H)** Representative images from muscle (soleus and EDL, respectively) sections pseudo colored for the different fiber types are shown. Primary antibodies against MHC I (clone BA-F8) and IIa (clone sc-71) were probed in the same sections to detect type I (red) and type IIa (green) fibers and antibody for MHC IIb (clone BF-F3) was used in different sections for the detection of type IIb fibers (red), bar is 100 μm. **(I** and **J)** Fiber type distributions in soleus and EDL, respectively. **(K** and **L)** Evaluation of average cross-sectional area (CSA) for the soleus and EDL, respectively. Fibers from seven mice of each genotype were analyzed. Number of individual fibers in soleus measured: type I: 980 (WT), 1,116 (EK); IIA: 1,898 (WT), 1,980 (EK), IIb/x: 417 (WT), 407 (EK). Number of individual fibers in EDL measured: IIA: 612 (WT), 505 (EK), IIb/x: 1,965 (WT), 2,323 (EK). Values in bar graphs are presented as mean ± SEM, n numbers are indicated. *P <0.05, **P <0.01, and ***P <0.001.

The observed changes in activity dependent Ca^2+^ handling, Ca^2+^-dependent signaling and protein synthesis in muscle suggest that the EK mutation should alter muscle function. Although the ability to generate force (normalized to cross-sectional area) was not different between muscles of young (between eight and 12 weeks of age) WT and EK mice (Figure 7A and B), both the soleus and the EDL muscles of young (between eight and 12 weeks of age) EK mice displayed small, but significant increases in the rate of fatigue (Figure 7C and D). However, older mice (over nine months) show significant decreases in force generation in the soleus (Figure 7E) and further increases in fatigue in the EDL (Figure 7F). Changes in muscle force and fatigue could arise from changes in fiber type distribution. We found that the fraction of fast twitch type IIb fibers increased and type IIx fibers decreased in both soleus and EDL muscle (Figure 7G-J), but the soleus also showed a decrease in Type 1 fibers (Figure 7I). A decrease in fiber CSAs in all fiber types in both soleus and EDL was also observed in EK compared to WT mice (Figure 7K and L). We also used a type IIb specific antibody to separately determine the CSA of the type IIb fibers in EDL muscles and found a significant (*P* <0.001) decrease of approximately 25% (CSA_WT_ = 1,810 ± 12 μm^2^, n = 1112 fibers; CSA_EK_ = 1,370 ± 14 μm^2^, n = 898 fibers, each from three different mice). However, we found no significant differences in grip strength, voluntary monitored wheel running, or endurance running in young (eight to 12 weeks of age) EK compared to WT mice, suggesting that the effects of the EK mutation on fiber type distribution, muscle contractility, and fatigue are relatively mild in young mice but increase in severity with age.

## Discussion

Although acute changes in depolarization-induced contraction in low Ca^2+^ and in the presence of dihydropyridines were previously reported [42], the role of Ca^2+^ permeation through Ca_V_1.1 in skeletal muscle has largely been ignored because Ca^2+^ influx through this channel is not required for ECC [2,43]. We demonstrate that Ca^2+^ signaling in skeletal muscle is significantly altered by a permeation defect in Ca_V_1.1 (E1014K, EK). This mutation resulted in concurrent inhibition of multiple Ca^2+^-sensitive pathways. These signaling changes translated to significant effects on muscle contractile function, providing compelling evidence that Ca^2+^ binding and/or permeation via Ca_V_1.1 is important for maintaining optimal muscle physiology.

The question that arises is whether the amount of Ca^2+^ entering the muscle fiber through Ca_V_1.1 is adequate to directly drive these changes in intracellular Ca^2+^ handling during repetitive stimulation. If so, previous studies have either underestimated the magnitude and relative contribution of Ca^2+^ influx through Ca_V_1.1 during repetitive stimulation of muscle, or Ca_V_1.1 Ca^2+^ binding and permeation activates a pathway (such as CaMKII) that promotes Ca^2+^ entry through a second Ca^2+^ influx channel (such as ECCE, SOCE, or TRP). Another possibility is that Ca^2+^ binding to the selectivity filter triggers a conformational change in Ca_V_1.1 that drives autophosphorylation of CaMKII bound to the channel (such as the C-terminus). CaMKII, activated by this mechanism, would then modulate multiple Ca^2+^ handling and downstream signaling pathways (SR Ca^2+^ stores, Ras, and mTORC1). The activation of these pathways would then alter numerous aspects of muscle physiology including fatigue, muscle fiber CSA, type II fiber type specification, and protein synthesis. Indeed, we demonstrate that autophosphorylation of CaMKII is decreased in the muscle of EK mice that have undergone repetitive stimulation, a condition that significantly enhances p-CaMKII levels in WT muscle.

The activation of different Ca^2+^-sensitive signaling pathways depends on the location, frequency, amplitude, and duration of the Ca^2+^ signal (reviewed by Tavi and Westerblad [16]). Important signaling molecules activated by Ca^2+^ use different combinations of these mechanisms. We demonstrate that CaMKII is localized close to Ca_V_1.1 and that the EK mutation decreases the proximity of CaMKII to Ca_V_1.1. Ca^2+^ permeation through Ca_V_1.1, together with CaMKII activation in WT fibers, slows the decline of the Ca^2+^ transient with repetitive stimulation by enhancing Ca^2+^ store refilling. This mechanism is deficient in fibers from EK mice. CaMKII activation is decreased when Ca^2+^ cannot permeate Ca_V_1.1, suggesting that Ca^2+^ binding within and/or movement through the Ca_V_1.1 pore is important for activation of CaMKII. Reciprocally, CaMKII appears to amplify Ca^2+^ influx (either through Ca_V_1.1 or a closely associated CaMKII-sensitive Ca^2+^ entry channel), since Ca^2+^ store refilling is decreased by CaMKII inhibitors in WT, but not EK fibers. However, at this point it is unclear whether the observed reduction in activity-dependent SR Ca^2+^ store content in FDB fibers from EK mice is due to loss of Ca_V_1.1 Ca^2+^ influx or a downstream effect of CaMKII activation on SR Ca^2+^ reuptake and/or store-operated Ca^2+^ entry.

One of the major findings in this study was a substantial decrease in insulin-mediated protein synthesis in EK muscle, as assessed by puromycin labelling and immunoblot analysis of the mTOR signaling pathway. The mTORC1 pathway activation in response to electrical stimulation is also decreased in EK compared to WT muscle. Decreased protein synthesis in response to growth factors such as insulin and repetitive stimulation may underlie the observed decrease in muscle fiber size in EK muscle. Activation of downstream substrates of mTORC1 was significantly decreased in EK muscle, as was activation of the upstream regulators of mTOR, Akt, and ERK1/2. We suggest that the Ca_V_1.1/CaMKII-dependent event that modulates insulin-mediated protein synthesis is upstream of PDK1 and/or mTORC2 activation, since phosphorylation of both Akt T308 (a PDK1 target) and Akt-S473 (an mTORC2 target) [44,45] are decreased in EK muscle following insulin treatment. This hypothesis is supported by previous studies that have reported Ca^2+^-dependent regulation of the PI3K/Akt/mTOR and Ras/Raf/ERK pathways [35-39].

The phenotypic changes associated with the EK mutation do not arise from any obvious changes in the neuromuscular junction. Chen *et al*. [9] found that a decrease in Ca^2+^ influx associated with a deficiency in the β-subunit of Ca_V_1.1 altered the formation of the neuromuscular junction. This may reflect a recently identified transcriptional role for the β1a-subunit [46], as opposed to its more conventional role in controlling Ca_V_1.1 Ca^2+^ currents, since mice with the EK mutation in Ca_V_1.1 lack Ca^2+^ currents but do not exhibit alterations in neuromuscular junction preformatting (Additional file 4: Figure S3). We also did not detect any changes in immune function (Additional file 5: Figure S4).

## Conclusions

While the magnitude of Ca^2+^ permeation through Ca_V_1.1 is small, there is a significant impact of this pathway on muscle function. Changes include increased fatigue, decreased muscle fiber diameter, increased type IIb fiber specification, and decreased protein synthesis. Thus, our results indicate that small changes in Ca_V_1.1 Ca^2+^ permeation, and CaMKII activation are amplified by changes in the activity of multiple critical downstream signaling pathways that impact a broad range of skeletal muscle functions. We propose that Ca^2+^ permeation through Ca_V_1.1, while not required for ECC, modulates multiple downstream Ca^2+^-sensitive signaling pathways to improve muscle function.

## Additional files

Additional file 1: Table S1. Antibodies used. Additional file 2: Figure S1. Creation of EK mice. Additional file 3: Figure S2. Effects of electrical stimulation. Additional file 4: Figure S3. EK mutation does not alter neuromuscular junction (NMJ) pre-patterning (A-F). Additional file 5: Figure S4. Analysis of the immune phenotype did not identify any significant differences between EK and WT mice.

## Abbreviations

4CmC: 4-chloro-m-cresol
9-AC: anthracene-9-carboxylic acid
AIP: autocamtide 2-related inhibitory peptide
BTS: N-benzyl-p-toluene sulfonamide
CaMKII: calmodulin-dependent protein kinase II
Ca_V_1.1: L-type voltage-dependent calcium release channel
CSA: cross-sectional area
CSQ: calsequestrin
ECC: excitation-contraction coupling
ECCE: excitation-coupled calcium entry
EDL: extensor digitorum longus
EK: mice with a glutamate to lysine mutation in the position 1014 of Ca_V_1.1
ETC: excitation-transcription coupling
FDB: flexor digitorum brevis
Fura 2 AM: fura-2 acetoxymethyl ester
GAPDH: Glyceraldehyde 3-phosphate dehydrogenase
HEPES: 4-(2-hydroxyethyl)-1-piperazineethanesulfonic acid
KRS: Krebs Ringer solution
MH: malignant hyperthermia
MHCI: myosin heavy chain I
MHCIIa: myosin heavy chain IIa
MHCIIb: myosin heavy chain IIb
NMJ: neuromuscular junction
NP-40: Tergitol-type NP-40
OCT: optimal cutting temperature
p: indicates phospho form if in front of a protein name
PBS: phosphate buffered saline
PLA: proximity ligation assay
PMSF: phenylmethylsulfonyl fluoride
PVDF: polyvinylidene difluoride
RBD: Ras binding domain
RyR1: ryanodine receptor 1
SDS: Sodium dodecyl sulfate
SERCA: Sarco/endoplasmic reticulum calcium ATPase
SOCE: store operated calcium entry
SR: sarcoplasmic reticulum
TEA-OH: tetraethylammonium hydroxide
TRP: Transient receptor potential
WT: wild type

## References

[1] Chandler WK, Rakowski RF, Schneider MF (1976). A non-linear voltage dependent charge movement in frog skeletal muscle. J Physiol.

[2] Dirksen RT, Beam KG (1999). Role of calcium permeation in dihydropyridine receptor function. insights into channel gating and excitation-contraction coupling. J Gen Physiol.

[3] Catterall WA (2011). Voltage-gated calcium channels. Cold Spring Harb Perspect Biol.

[4] Stroffekova K (2008). Ca2+/CaM-dependent inactivation of the skeletal muscle L-type Ca2+ channel (CaV1.1). Pflügers Archiv. Euro J Physiol.

[5] Garcia J, Tanabe T, Beam KG (1994). Relationship of calcium transients and charge movements in myotubes expressing skeletal and cardiac dihydropyridine receptors. J Gen Physiol.

[6] Zhou J, Yi J, Royer L, Launikonis BS, González A, García J, Ríos E (2006). A probable role of dihydropyridine receptors in repression of Ca^2+^ sparks demonstrated in cultured mammalian muscle. Am J Physiol Cell Physiol.

[7] Rotzler S, Schramek H, Brenner HR (1991). Metabolic stabilization of endplate acetylcholine receptors regulated by Ca^2^+ influx associated with muscle activity. Nature.

[8] Caroni PS, Rotzler BJC, Brenner HR (1993). Calcium influx and protein phosphorylation mediate the metabolic stabilization of synaptic acetylcholine receptors in muscle. J Neurosci.

[9] Chen F, Liu Y, Sugiura Y, Allen PD, Gregg RG, Lin W (2011). Neuromuscular synaptic patterning requires the function of skeletal muscle dihydropyridine receptors. Nat Neurosci.

[10] Jorquera G, Altamirano F, Contreras-Ferrat A, Almarza G, Buvinic S, Jacquemond V, Jaimovich E, Casas M (2013). Cav1.1 controls frequency-dependent events regulating adult skeletal muscle plasticity. J Cell Sci.

[11] Vandebrouck C, Martin D, Colson-Van Schoor M, Debaix H, Gailly P (2002). Involvement of TRPC in the abnormal calcium influx observed in dystrophic (mdx) mouse skeletal muscle fibers. J Cell Biol.

[12] Friedrich O, von Wegner F, Chamberlain JS, Fink RH, Rohrbach P (2008). L-type Ca^2+^ channel function is linked to dystrophin expression in mammalian muscle. PLoS One.

[13] Tang ZZ, Yarotskyy V, Wei L, Sobczak K, Nakamori M, Eichinger K, Moxley RT, Dirksen RT, Thornton CA (2012). Muscle weakness in myotonic dystrophy associated with misregulated splicing and altered gating of Ca(V)1.1 calcium channel. Hum Mol Genet.

[14] Cherednichenko G, Ward CW, Feng W, Cabrales E, Michaelson L, Samso M, López JR, Allen PD, Pessah IN (2008). Enhanced excitation-coupled calcium entry in myotubes expressing malignant hyperthermia mutation R163C is attenuated by dantrolene. Mol Pharmacol.

[15] Eltit JM, Bannister RA, Moua O, Altamirano F, Hopkins PM, Pessah IN, Molinski TF, López JR, Beam KG, Allen PD (2012). Malignant hyperthermia susceptibility arising from altered resting coupling between the skeletal muscle L-type Ca^2+^ channel and the type 1 ryanodine receptor. Proc Natl Acad Sci U S A.

[16] Tavi P, Westerblad H (2011). The role of in vivo Ca^2+^ signals acting on Ca^2+^-calmodulin-dependent proteins for skeletal muscle plasticity. J Physiol.

[17] Hudmon A, Schulman H, Kim J, Maltez JM, Tsien RW, Pitt GS (2005). CaMKII tethers to L-type Ca^2+^ channels, establishing a local and dedicated integrator of Ca^2+^ signals for facilitation. J Cell Biol.

[18] Abiria SA, Colbran RJ (2010). CaMKII associates with CaV1.2 L-type calcium channels via selected beta subunits to enhance regulatory phosphorylation. J Neurochem.

[19] Ma H, Cohen S, Li B, Tsien RW (2012). Exploring the dominant role of Cav1 channels in signalling to the nucleus. Biosci Rep.

[20] Zhang P, Li MZ, Elledge SJ (2002). Towards genetic genome projects: genomic library screening and gene-targeting vector construction in a single step. Nat Genet.

[21] Tallquist MD, Soriano P (2000). Epiblast-restricted Cre expression in MORE mice: a tool to distinguish embryonic vs. extra-embryonic gene function. Genesis.

[22] Beam KG, Knudson CM (1988). Calcium currents in embryonic and neonatal mammalian skeletal muscle. J Gen Physiol.

[23] Butler M, McKay RA, Popoff IJ, Gaarde WA, Witchell D, Murray SF, Dean NM, Bhanot S, Monia BP (2002). Specific inhibition of PTEN expression reverses hyperglycemia in diabetic mice. Diabetes.

[24] Goodman CA, Mabrey DM, Frey JW, Miu MH, Schmidt EK, Pierre P, Hornberger TA (2011). Novel insights into the regulation of skeletal muscle protein synthesis as revealed by a new nonradioactive in vivo technique. FASEB J.

[25] Schmidt EK, Clavarino G, Ceppi M, Pierre P (2009). SUnSET, a nonradioactive method to monitor protein synthesis. Nat Methods.

[26] Schertzer JD, Green HJ, Fowles JR, Duhamel TA, Tupling AR (2004). Effects of prolonged exercise and recovery on sarcoplasmic reticulum Ca^2+^ cycling properties in rat muscle homogenates. Acta Physiol Scand.

[27] Gehrig SM, van der Poel C, Sayer TA, Schertzer JD, Henstridge DC, Church JE, Lamon S, Russell AP, Davies KE, Febbraio MA, Lynch GS (2012). Hsp72 preserves muscle function and slows progression of severe muscular dystrophy. Nature.

[28] Sakamoto K, Hirshman MF, Aschenbach WG, Goodyear LJ (2002). Contraction regulation of Akt in rat skeletal muscle. J Biol Chem.

[29] Yang J, Ellinor PT, Sather WA, Zhang JF, Tsien RW (1993). Molecular determinants of Ca^2+^ selectivity and ion permeation in L-type Ca^2+^ channels. Nature.

[30] Bannister RA, Beam KG (2011). Properties of Na^+^ currents conducted by a skeletal muscle L-type Ca^2+^ channel pore mutant (SkEIIIK). Channels (Austin).

[31] Cherednichenko G, Hurne AM, Fessenden JD, Lee EH, Allen PD, Beam KG, Pessah IN (2004). Conformational activation of Ca^2+^ entry by depolarization of skeletal myotubes. Proc Natl Acad Sci U S A.

[32] Bannister RA, Pessah IN, Beam KG (2009). The skeletal L-type Ca^2+^ current is a major contributor to excitation-coupled Ca^2+^ entry. J Gen Physiol.

[33] Yang E, Schulman H (1999). Structural examination of autoregulation of multifunctional calcium/calmodulin-dependent protein kinase II. J Biol Chem.

[34] Ishida A, Kameshita I, Okuno S, Kitani T, Fujisawa H (1995). A novel highly specific and potent inhibitor of calmodulin-dependent protein kinase II. Biochem Biophys Res Commun.

[35] Mercan F, Lee H, Kolli S, Bennett AM (2013). Novel role for SHP-2 in nutrient-responsive control of S6 kinase 1 signaling. Mol Cell Biol.

[36] Graves LM, He Y, Lambert J, Hunter D, Li X, Earp HS (1997). An intracellular calcium signal activates p70 but not p90 ribosomal S6 kinase in liver epithelial cells. J Biol Chem.

[37] Hannan KM, Thomas G, Pearson RB (2003). Activation of S6K1 (p70 ribosomal protein S6 kinase 1) requires an initial calcium-dependent priming event involving formation of a high-molecular-mass signalling complex. Biochem J.

[38] Gulati P, Gaspers LD, Dann SG, Joaquin M, Nobukuni T, Natt F, Kozma SC, Thomas AP, Thomas G (2008). Amino acids activate mTOR complex 1 via Ca^2+^/CaM signaling to hVps34. Cell Metab.

[39] Conus NM, Hemmings BA, Pearson RB (1998). Differential regulation by calcium reveals distinct signaling requirements for the activation of Akt and p70S6k. J Biol Chem.

[40] Yang Q, Inoki K, Kim E, Guan KL (2006). TSC1/TSC2 and Rheb have different effects on TORC1 and TORC2 activity. Proc Natl Acad Sci U S A.

[41] Salzano M, Rusciano MR, Russo E, Bifulco M, Postiglione L, Vitale M (2012). Calcium/calmodulin-dependent protein kinase II (CaMKII) phosphorylates Raf-1 at serine 338 and mediates Ras-stimulated Raf-1 activation. Cell Cycle.

[42] Dulhunty AF, Gage PW (1988). Effects of extracellular calcium concentration and dihydropyridines on contraction in mammalian skeletal muscle’. J Physiol.

[43] Armstrong CM, Bezanilla FM, Horowicz P (1972). Twitches in the presence of ethylene glycol bis(-aminoethyl ether)-N, N’-tetracetic acid. Biochim Biophys Acta.

[44] Alessi DR, James SR, Downes CP, Holmes AB, Gaffney PR, Reese CB, Cohen P (1997). Characterization of a 3-phosphoinositide-dependent protein kinase which phosphorylates and activates protein kinase Balpha. Curr Biol.

[45] Sarbassov DD, Guertin DA, Ali SM, Sabatini DM (2005). Phosphorylation and regulation of Akt/PKB by the rictor-mTOR complex. Science.

[46] Taylor J, Pereyra A, Zhang T, Messi ML, Wang ZM, Hereñú C, Kuan PF, Delbono O (2014). The Cavβ1a subunit regulates gene expression and suppresses myogenin in muscle progenitor cells. J Cell Biol.

