# Supplementary material for: Ca2+ permeation and/or binding to CaV1.1 fine-tunes skeletal muscle Ca2+ signaling to sustain muscle function
Source: Skelet Muscle. 2015 Jan 29;5:4. doi: 10.1186/s13395-014-0027-1 (PMC4340672; doi:10.1186/s13395-014-0027-1)
Supplement: Additional file 1: Table S1. — Antibodies used. [file 13395_2014_27_MOESM1_ESM.pdf]

Supplemental Table 1: Antibodies used

Antibody	Company	WB	IHC	P.L.A.	Cat. No.
$Ca_v1.1\beta$	Santa Cruz			1:30	sc-15970
$Ca_v1.1\alpha$	Thermo Scientific	1:1000	1:200		MA3-920
CaMKII	Santa Cruz			1:50	sc-9035
CaMKII	Cell Signaling	1:1000	1:100		#3362
Phospho CaMKII (T286)	Cell Signaling	1:1000			#3361
Calsequestrin	Thermo Scientific	1:1000			PA1-913
RyR1	Thermo Scientific	1:2000			MA3-925
SERCA1	Thermo Scientific	1:1000			MA3-911
SERCA2	Thermo Scientific	1:1000			MA3-919
Sarcolipin	ProteinTech Group	1:1000			183951-1-AP
GAPDH	Santa Cruz	1:500			sc-20357
GSK3β	Santa Cruz	1:500			sc-9166
Phospho GSK3β (S9)	Santa Cruz	1:500			sc-11757
Phospho Akt1 (S473)	Cell Signaling	1:1000			#9271
Phospho Akt2 (S474)	Thermo Scientific	1:1000			PA1-14034
Phospho Akt1 (T308)	Cell Signaling	1:1000			#2965P
Akt1/2	Santa Cruz	1:500			sc-1619
Neurofilament M	Millipore		1:200		AB1987
Myosin Heavy Chain 1	DSHB		1:50		BA-F8
Myosin Heavy Chain 2a	DSHB		1:50		SC-71
Myosin Heavy Chain 2b	DSHB		1:50		BF-F3
Phospho-eEF2 (T56)	Cell Signaling	1:1000			#2331
eEF2	Cell Signaling	1:1000			#2332
Phospho-S6 (S235/236)	Cell Signaling	1:1000			#4858
S6	Cell Signaling	1:1000			#2317
Phospho-4E-BP1 (T37/47)	Cell Signaling	1:1000			#2855
4E-BP1	Cell Signaling	1:1000			#9644
Phospho ERK (T202/Y204)	Santa Cruz	1:500			sc-16982-R
Phospho mTOR (S2844)	Cell Signaling	1:1000			#2971S
mTOR	Cell Signaling	1:1000			#2972
Puromycin	KeraFAST	1:1000			EQ-0001
Phospho Raf (S338)	Cell Signaling	1:1000			#9427
Raf-1	Santa Cruz	1:500			sc-227
Ras	abcam	1:1000			ab79973

WB=Western blotting, IHC=Immunohistochemistry, P.L.A.=Proximity Ligation Assay

Abcam (Cambridge, MA, United States); Cell Signaling (Danvers, MA, United States); DSHB (Iowa City, United States); KeraFAST (Boston, MA, United States); Millipore (Billerica, Massachusetts, United States); ProteinTech group (Chicago, IL, United States); Santa Cruz (Santa Cruz, CA, United States); Thermo Scientific (Waltham, MA, United States)
