# Supplementary figures and images for: Ca2+ permeation and/or binding to CaV1.1 fine-tunes skeletal muscle Ca2+ signaling to sustain muscle function
Source: Skelet Muscle. 2015 Jan 29;5:4. doi: 10.1186/s13395-014-0027-1 (PMC4340672; doi:10.1186/s13395-014-0027-1)

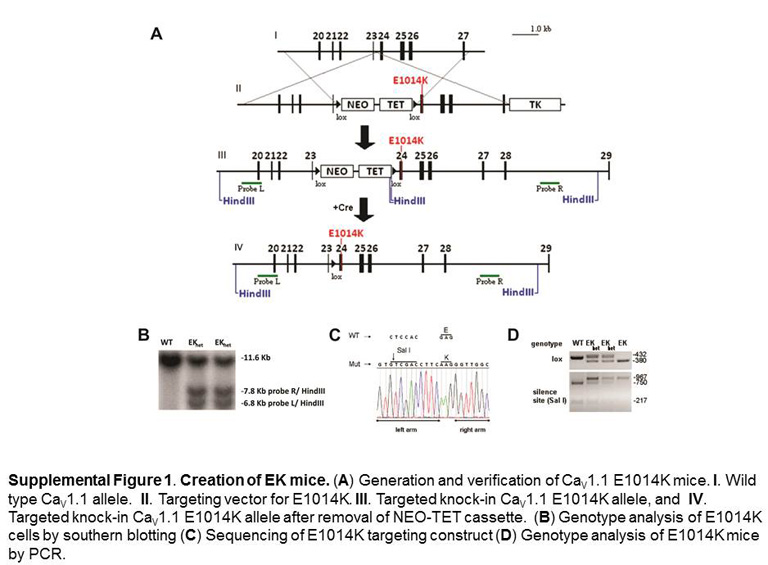

Supplement: Additional file 2: Figure S1. — Creation of EK mice. [file 13395_2014_27_MOESM2_ESM.jpeg]

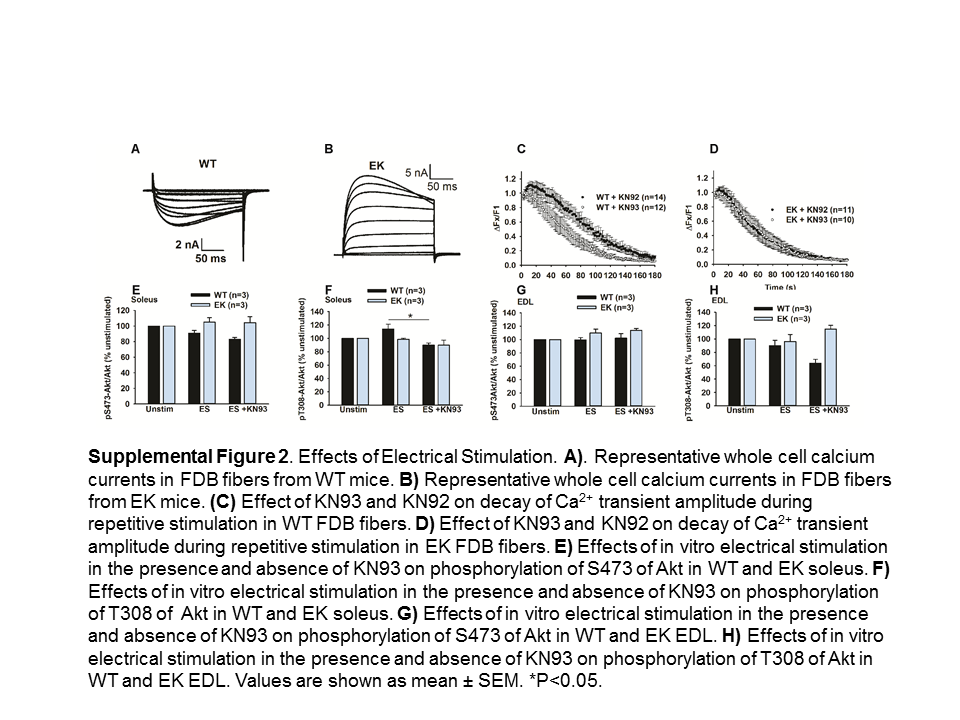

Supplement: Additional file 3: Figure S2. — Effects of electrical stimulation. [file 13395_2014_27_MOESM3_ESM.tif]

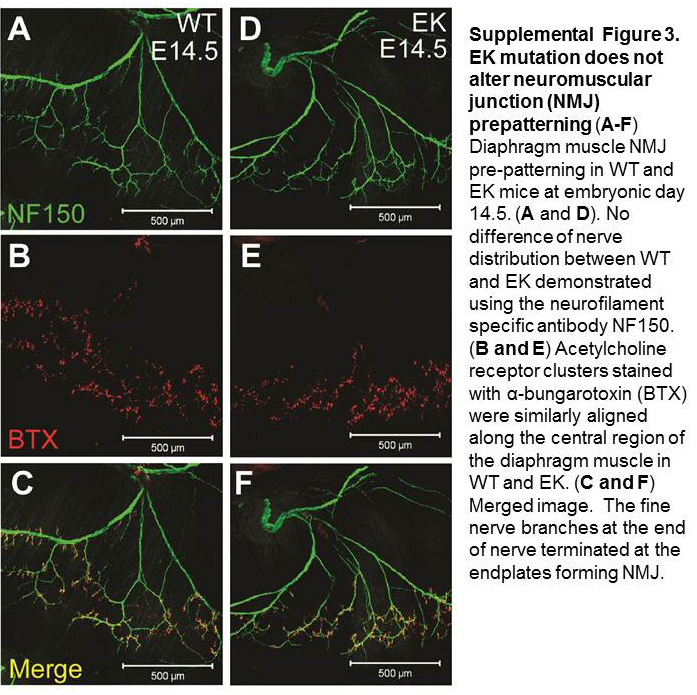

Supplement: Additional file 4: Figure S3. — EK mutation does not alter neuromuscular junction (NMJ) pre-patterning (A-F). [file 13395_2014_27_MOESM4_ESM.jpeg]

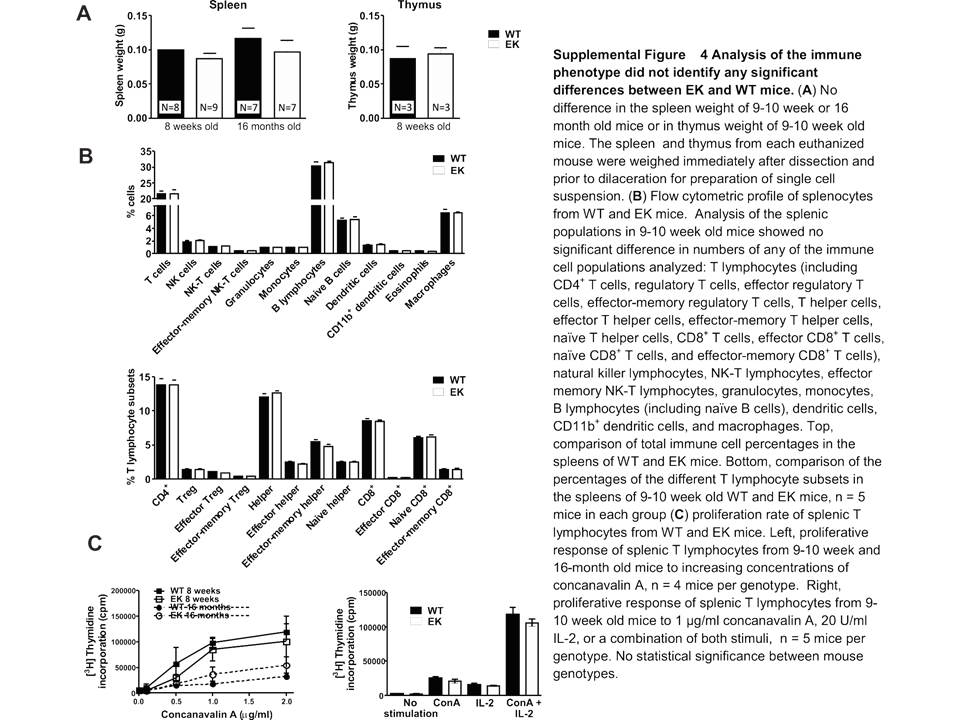

Supplement: Additional file 5: Figure S4. — Analysis of the immune phenotype did not identify any significant differences between EK and WT mice. [file 13395_2014_27_MOESM5_ESM.jpeg]
